# Predictors of referral for fertility preservation counseling in patients diagnosed with cancer

**DOI:** 10.1007/s10815-025-03784-z

**Published:** 2026-01-02

**Authors:** Paris N. Chey, Miranda L. M. Delwalla, Julia Shuford, Natalie Albright, Katharina Goebel, Julia Grigorian, Sheila Rajagopalan, D. Austin Schirmer, Heather S. Hipp

**Affiliations:** 1 Emory University School of Medicine, Atlanta, GA USA; 2 Division of Research, Department of Gynecology and Obstetrics, Emory University School of Medicine, Atlanta, GA USA; 3 Division of Reproductive Endocrinology and Infertility, Department of Gynecology and Obstetrics, Emory University School of Medicine, Atlanta, GA USA; 4 Department of Obstetrics and Gynecology, Pennsylvania Hospital, Philadelphia, PA USA

**Keywords:** Oncofertility, Reproductive health, Fertility preservation, Survivorship, Cancer

## Abstract

**Objective:**

Fertility preservation (FP) is an important aspect of care for reproductive-aged cancer patients since treatment can significantly impact fertility. Despite professional recommendations for early referral to FP, rates remain low. This study evaluates predictors of FP referrals by Oncologists to Reproductive Endocrinology and Infertility (REI) physicians among reproductive-aged cancer patients at a large academic institution.

**Materials and methods:**

This cross-sectional study evaluated patients ages 14–42 years seen by an Oncologist from 2022 to 2024 with a new diagnosis of cancer for whom planned treatment is either gonadotoxic or requires delayed pregnancy. Referral rates to REI physicians were assessed by patient demographics and cancer and Oncologist characteristics as potential predictors. Statistical analysis used chi-square tests, Fisher’s exact tests, and multivariable logistic regression.

**Results:**

Of 510 patients seen by an Oncologist for a newly diagnosed cancer, only 127 (22.5%) were referred for FP. Patients > 30 years were less likely to be referred than younger patients (aOR = 0.15, 95% CI = 0.05, 0.47, *p* = 0.001). Patients with breast cancer were most likely to be referred compared to other cancers. There was no difference in referral rates by physician gender. Physicians with 21–30 years of experience were more likely to refer than those with < 10 years (aOR = 2.89, 95% CI = 1.50, 5.62, *p* = 0.002).

**Conclusions:**

There was a low rate of FP referral and significant disparities based on patient demographics and clinical factors. Referral rates were higher among younger patients and breast cancer patients. Standardizing FP referrals across oncology specialties, consideration of electronic health record “soft stops” for referral and increasing patient and provider education are essential to improving referral rates.

## Introduction

Oncology patients of reproductive age face the dual challenge of fighting cancer and mitigating long-term negative effects of treatment, including infertility [1]. While cancer treatment has resulted in the improvement of 5-year survival rates since the 1970 s, 40–80% of reproductive-aged female cancer survivors will experience infertility or premature ovarian insufficiency (POI) [2]. Thus, fertility preservation (FP), including oocyte and sperm cryopreservation, is a crucial piece of care for patients of reproductive age to minimize the impact of cancer treatment on reproductive function [1]. The American Society of Clinical Oncology (ASCO) recommends thorough FP discussions with patients before and at several steps throughout cancer treatment [3].

Despite the acknowledged importance of FP in oncology care, current care models have barriers to optimal implementation [4–7]. FP referral rates range from 10 to 40% [1, 2, 8–10], though 60% serves as a promising benchmark according to prior literature [11]. Barriers to referral include provider knowledge gaps, inconsistent interdisciplinary collaboration, and inadequate patient education [12–15]. Alongside weaknesses in the healthcare system, patient-specific factors contribute to disparities in FP. Lesbian, pansexual, and queer patients have less FP discussions compared to bisexual patients [16]. Another known barrier is patient geographic distance to fertility clinics [17].

There is limited research examining the effects of provider characteristics and patient-specific factors as predictors of FP referrals [9, 18]. We aimed to define potential predictors of referral and non-referral, including patient and provider demographics in a diverse group of reproductive-aged patients with different types of cancer at an urban southeastern academic center. We also evaluated referral rates based on types of treatment, classified as gonadotoxic treatment and teratogenic treatment.

## Materials and methods

**Fig. 1:**
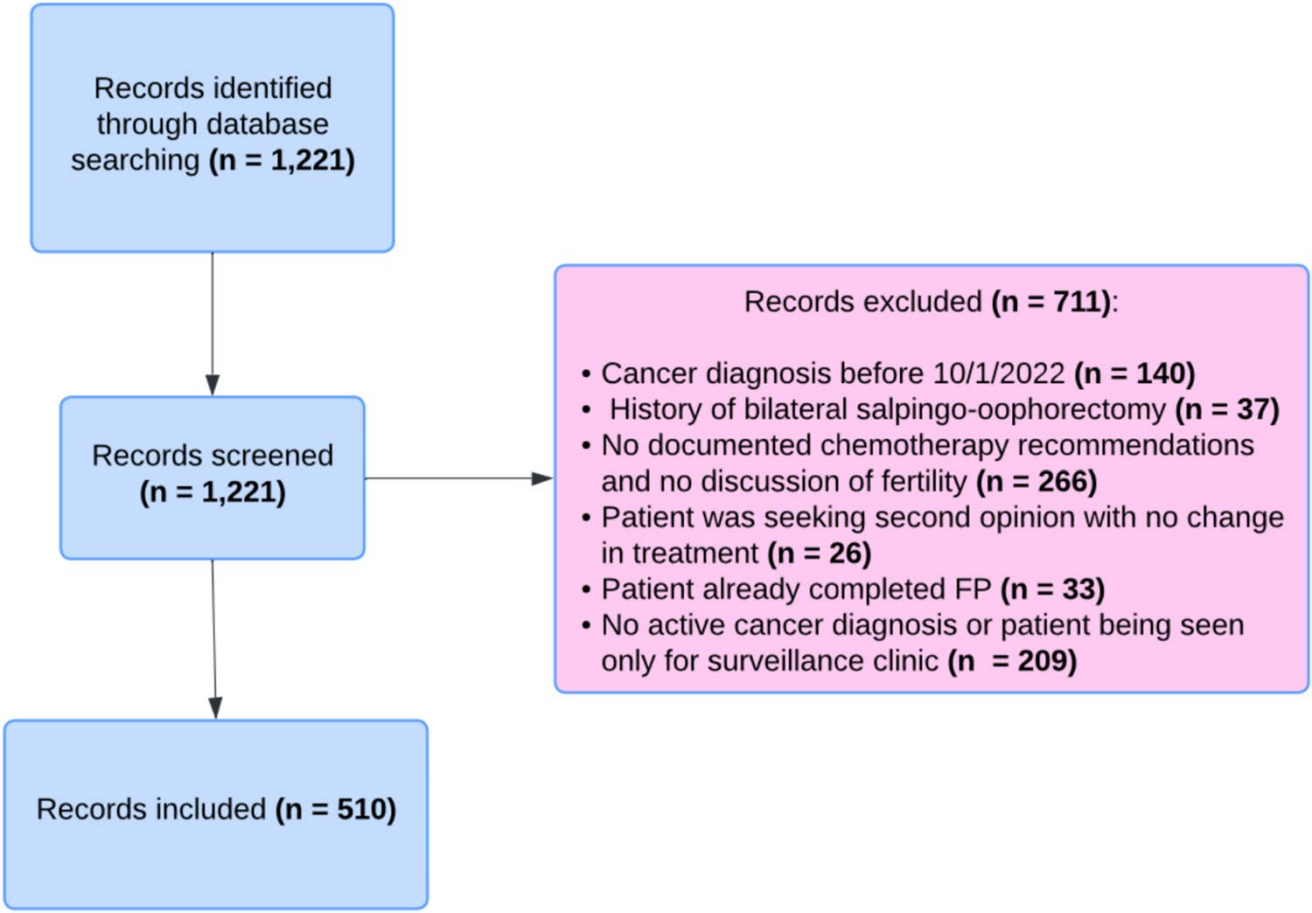
Flowchart depicting the inclusion and exclusion criteria for patients in this study

This cross-sectional study was conducted at a southeastern academic center during a 2-year period (October 1, 2022, to October 1, 2024). This study was reviewed and obtained IRB approval. Eligible patients were identified based on the following criteria: reproductive age (14–42 years old at time of initial visit), an initial oncology appointment billing code in the electronic health record (EHR), and a diagnosis of cancer. Patients also required a potential plan to receive treatment that was either gonadotoxic and/or teratogenic (defined below). From the initial cohort of 1221 patients, 711 patients were excluded (Fig. 1). The remaining 510 patients constituted the analytic sample. Referrals from Oncology to Reproductive Endocrinology & Infertility (REI) physicians within the same academic institution were placed through an order within the EHR. The referrals were generated based on oncologist discretion. Patients were generally seen by REI physicians within 1 week of their referral.

Patient demographics extracted from the EHR included biological sex, self-described race and ethnicity, occupation, insurance status, and relationship status. Other variables collected included parity (specifically, number of living children amongst female patients only), oncology information (type of cancer, prior chemotherapy treatment), and presence of a documented fertility discussion. Cancer types in the analytic sample were breast, lymphoma/leukemia, sarcoma, gynecologic cancer (ovarian, cervical, uterine), and an “other” category, which included small numbers of patients diagnosed with colon cancer, melanoma, aplastic anemia, and multiple myeloma. Information about Oncologists was gathered using the hospital website directory, including gender and years since residency graduation.

**Fig. 2:**
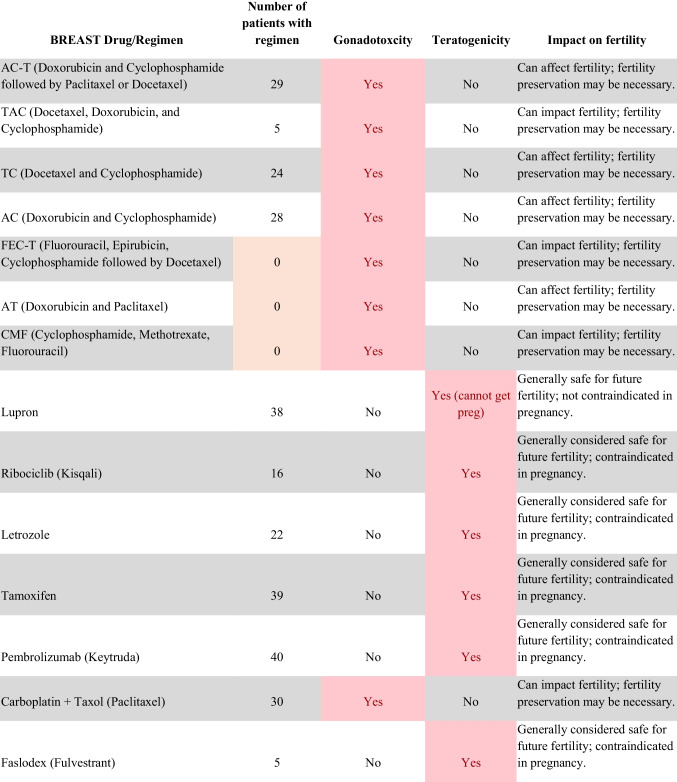

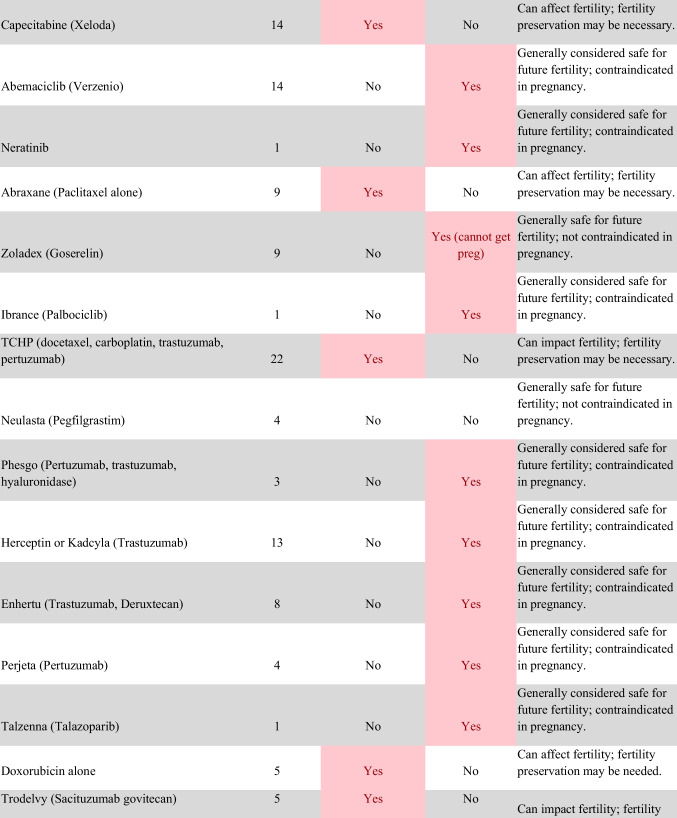

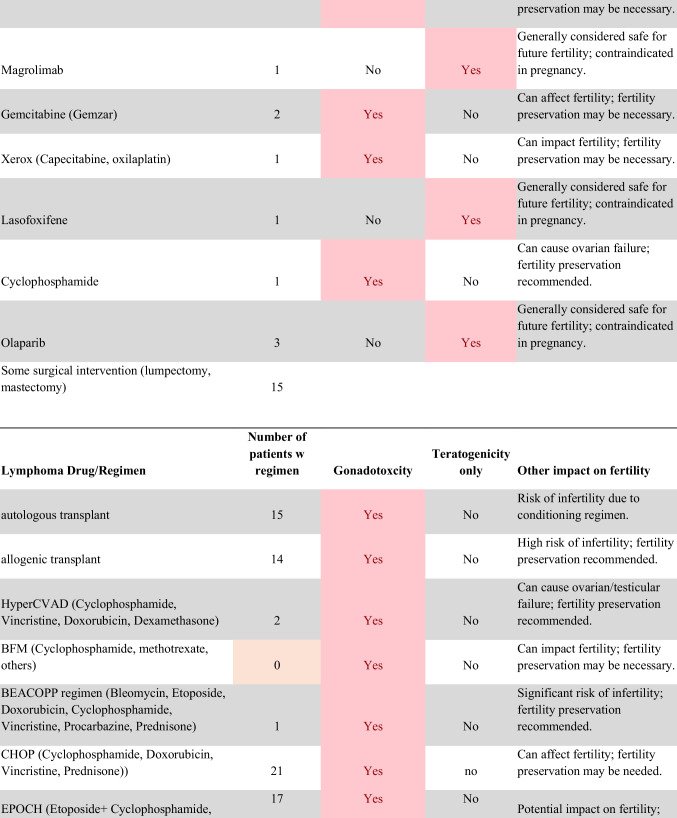

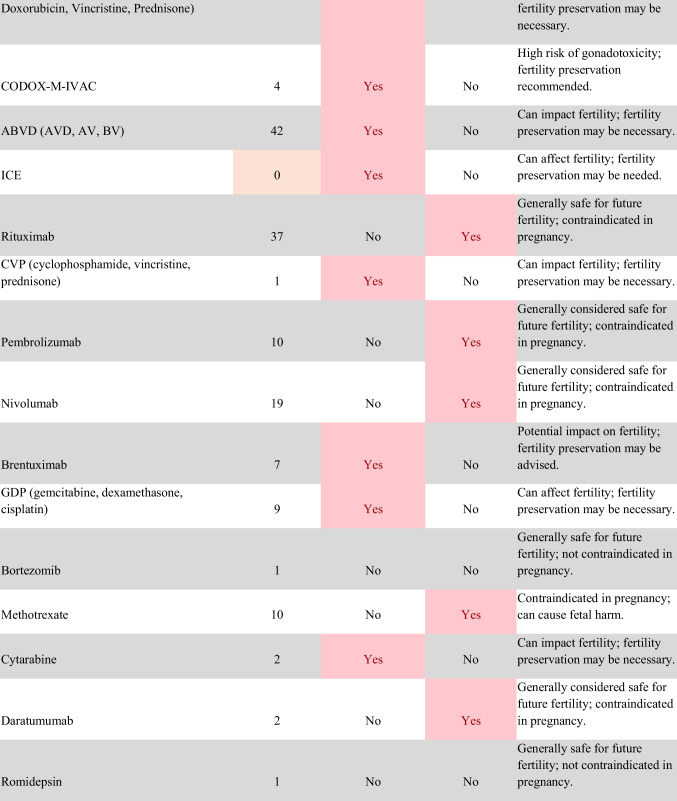

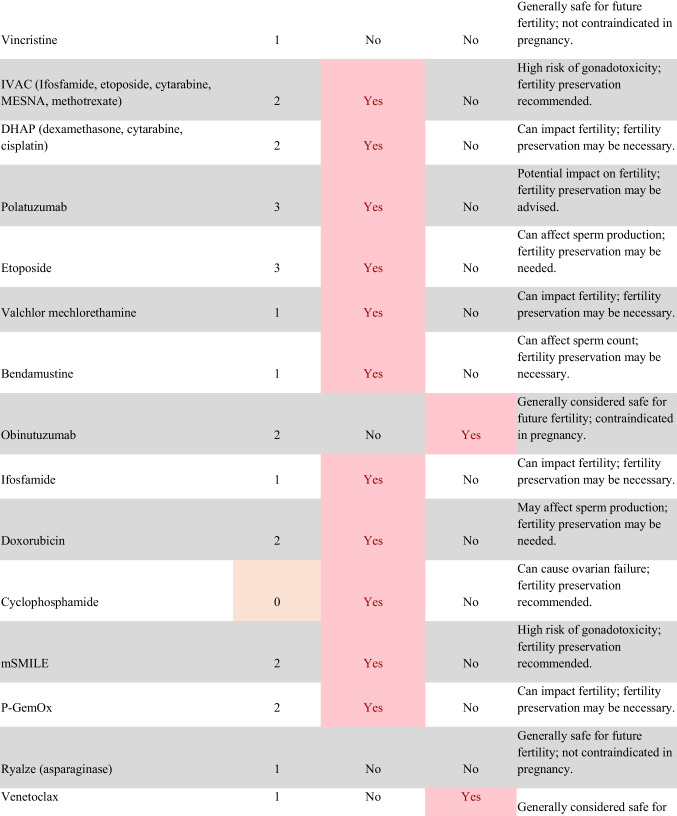

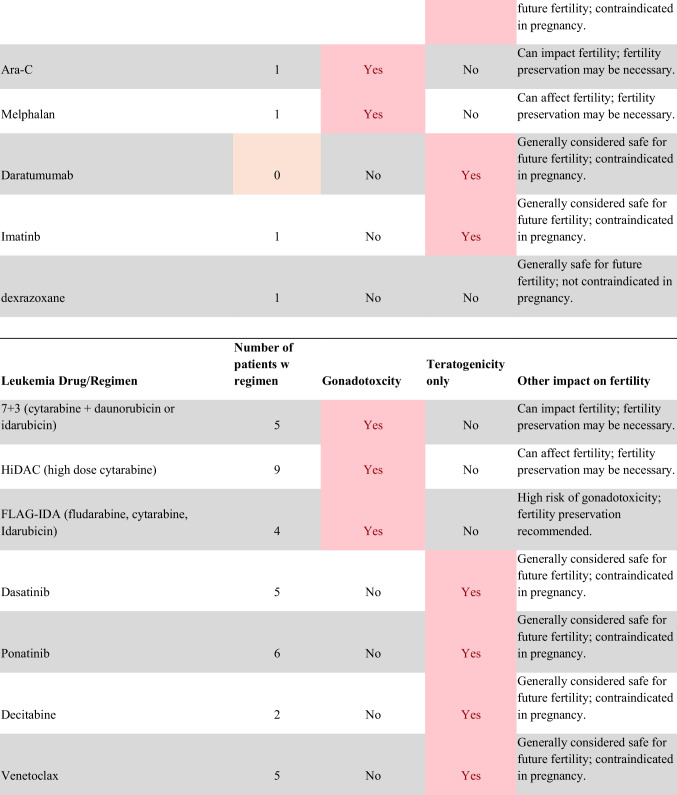

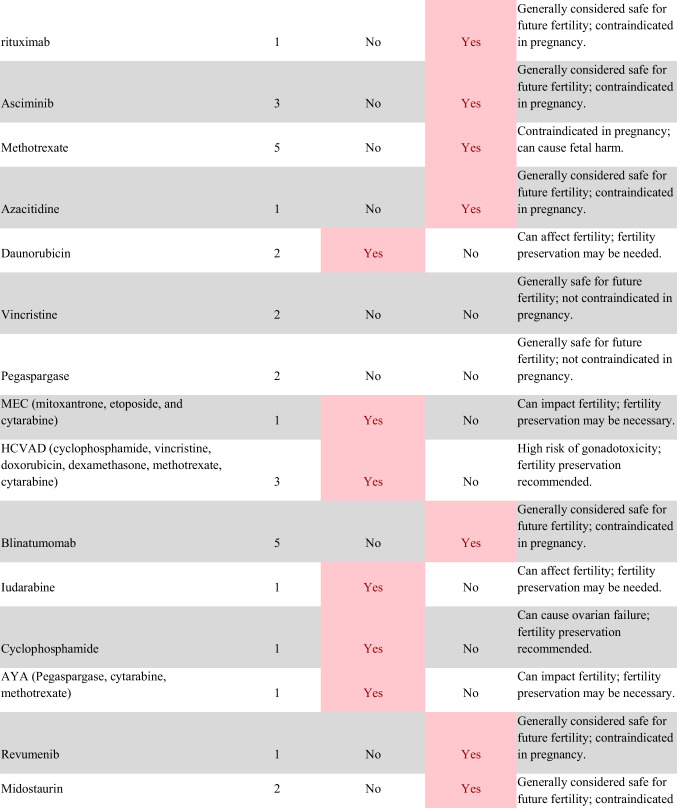

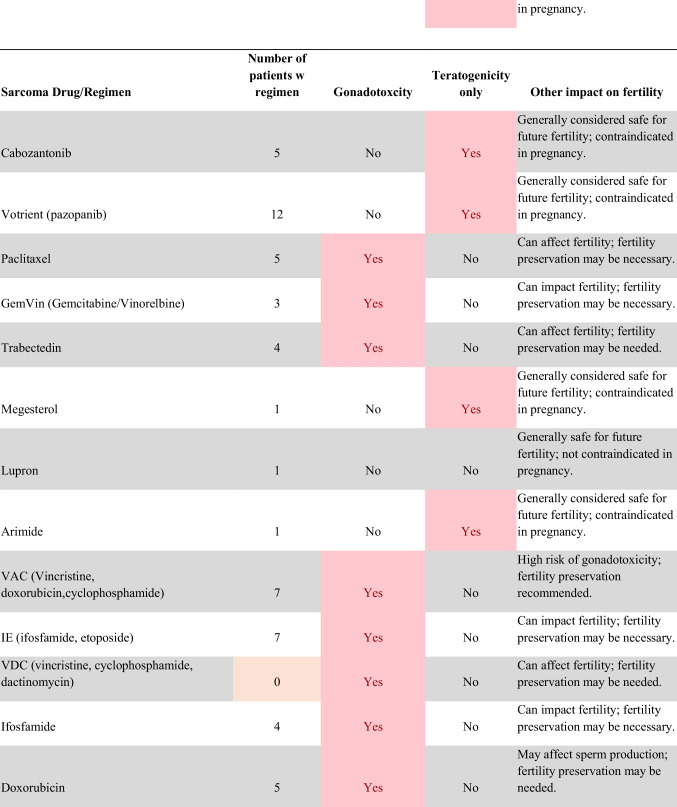

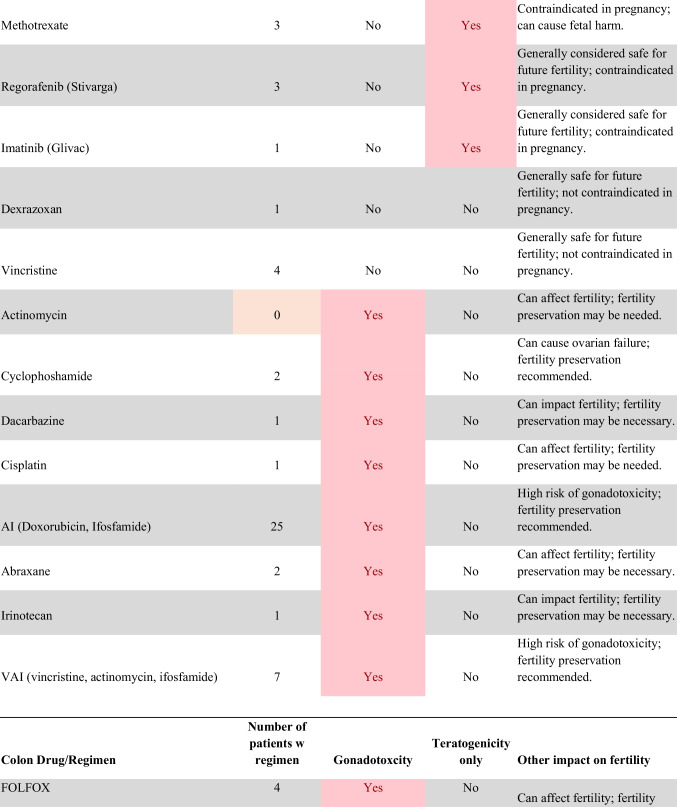

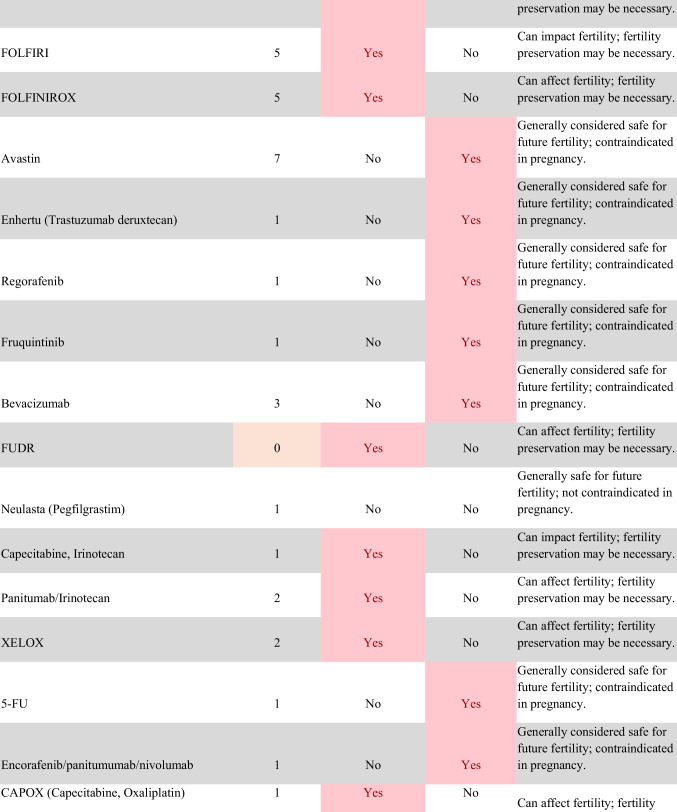

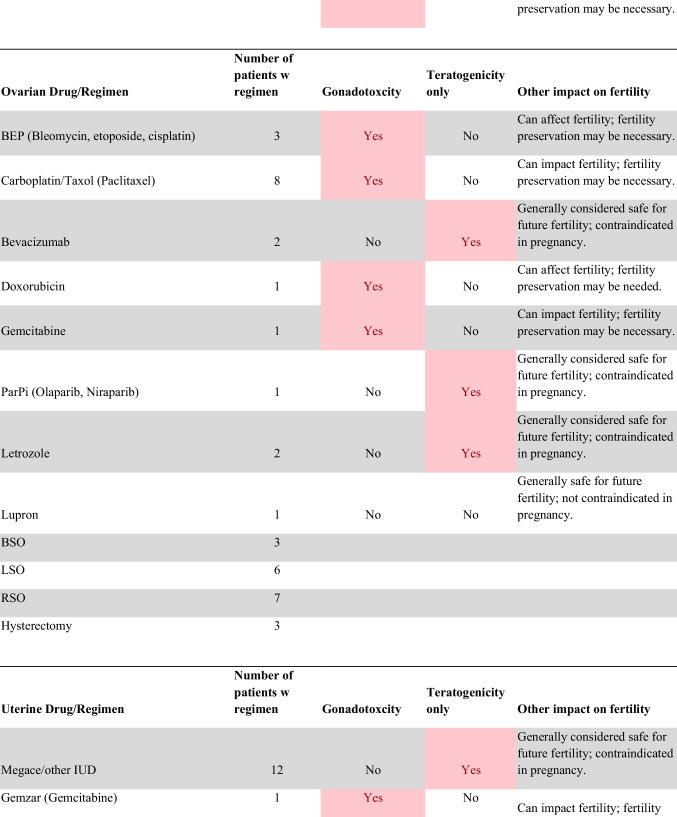

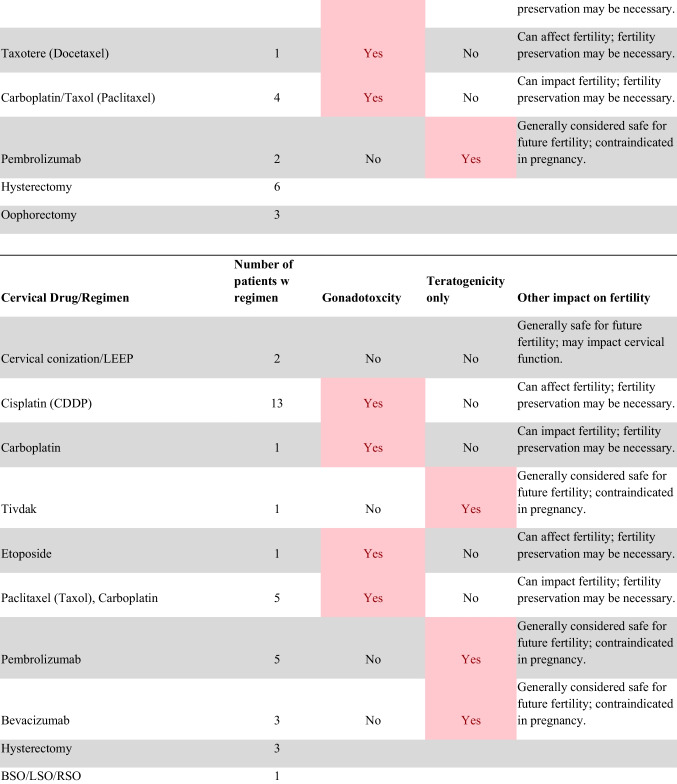
Classification of gonadotoxic and teratogenic drugs

Chemotherapeutic regimens were analyzed and classified into two primary categories: gonadotoxic and teratogenic therapy (Fig. 2). Gonadotoxic chemotherapy was defined as treatments with a significant threat to ovarian or testicular function and risk to fertility [19, 20]. For patients prescribed these drugs, FP is highly recommended prior to cancer treatment [21]. We classified drugs as teratogenic if they either prevent pregnancy or have a risk of teratogenicity. The required delay in pregnancy with these teratogenic medications can include multiple years [22], increasing the risk of age-related infertility in patients, thus warranting a FP referral to mitigate the potential fertility decline over time [23, 24]. Patients were classified as planning to receive gonadotoxic or teratogenic treatment if these medications were indicated and recommended in the EHR.

 To compare rates of referrals by clinical characteristics, variables were analyzed using frequencies, percentages, Pearson’s chi-square tests, and Fisher’s exact tests. Multivariable logistic regression was used to assess associations of patient demographics, cancer type, treatment type, and Oncologist characteristics with FP referral with adjusted odds ratios (aORs) and 95% confidence intervals (CIs). Statistical tests were assessed with a critical value of 0.05. Analyses were conducted in R version 4.3.3 (Figs. 1 and 2).

## Results

### Patient characteristics in study cohort

Of the 510 patients, 125 (22.5%) patients were referred and 385 (77.4%) patients were not referred by their oncologist to REI for counseling prior to oncologic treatment (Table 1). Approximately three-quarters (73.9%) identified as biologically female, and 26.1% as biologically male. Referral rates differed by biologic sex, with fewer female patients referred (female 22.5%; male 31.6%; *p* = 0.029). Of the ages included, patients aged 21–30 years were significantly the most referred for FP (44.1%), (compared to not referred 57.2%, *p* < 0.001). Most patients identified as non-Hispanic White (43.9%) or non-Hispanic Black (39.3%), with smaller proportions identifying as non-Hispanic Asian/Pacific Islander (10.5%) or Hispanic/Latino (6.3%). Referral rates did not differ by self-identified race/ethnicity (*p* = 0.502).

**Table 1:** Patient characteristics in study cohort

Characteristics	Total (*N* = 506)	Referred (*N* = 127)	Not referred (*N* = 379)	*p*-value
*Biological sex*				0.029
Female	374 (73.9%)	84 (22.5%)	290 (77.5%)	
Male	132 (26.1%)	43 (31.6%)	89 (67.4%)	
*Age (years)*				< 0.001*
14–20	17 (3.4%)	10 (58.8%)	7 (41.2%)	
21–30	143 (28.3%)	63 (44.1%)	83 (57.2%)	
31–40	302 (59.7%)	48 (15.9%)	258 (84.0%)	
41–42	42 (8.3%)	5 (11.9%)	37 (88.1%)	
*Race/ethnicity*				0.502
Hispanic	32 (6.3%)	11 (34.4%)	21 (65.6%)	
Non-Hispanic Asian/Pacific Islander	53 (10.5%)	11 (20.8%)	42 (79.2%)	
Non-Hispanic Black	199 (39.3%)	47 (23.6%)	152 (76.4%)	
Non-Hispanic White	222 (43.9%)	58 (26.1%)	164 (73.9%)	
*Occupation*				< 0.001
Administrative	22 (4.3%)	5 (22.7%)	17 (77.3%)	
Arts/media/sports	24 (4.7%)	5 (20.8%)	19 (79.2%)	
Business/finance/sales/management/legal	113 (22.3%)	23 (20.4%)	93 (80.9%)	
Computer/tech/architecture/engineering/science	39 (7.7%)	16 (41.0%)	23 (59.0%)	
Education	32 (6.3%)	6 (18.8%)	26 (81.3%)	
Healthcare	59 (11.7%)	21 (35.6%)	36 (64.3%)	
Service/construction/agriculture	110 (21.7%)	29 (26.4%)	85 (74.6%)	
Student	29 (5.7%)	13 (44.8%)	16 (55.2%)	
Unemployed	77 (15.2%)	8 (10.4%)	70 (89.7%)	
*Insurance status*				0.391*
Insured	487 (96.2%)	120 (24.6%)	367 (75.4%)	
Uninsured	17 (3.4%)	6 (35.3%)	11 (64.7%)	
*Types of insurance*				0.454*
Medicaid	63 (12.5%)	–-	–-	
Medicare	15 (3.1%)	–-	–-	
Private insurance	409 (80.4%)	105 (25.7%)	304 (74.3%)	
*Partner status*				0.003
In a relationship	46 (9.1%)	15 (32.6%)	31 (67.4%)	
Married	229 (45.3%)	41 (17.9%)	188 (82.1%)	
Single	231 (45.7%)	71 (30.7%)	160 (69.3%)	
*Type of cancer*				
Breast	200 (39.5%)	46 (23.0%)	154 (77.0%)	0.010
Gynecological cancer	74 (14.6%)	19 (25.7%)	55 (74.3%)	
Lymphoma/leukemia	141 (27.9%)	49 (34.8%)	92 (65.2%)	
Other	29 (5.7%)	5 (17.2%)	24 (82.8%)	
Sarcoma	62 (12.3%)	8 (12.9%)	54 (87.1%)	
*Living children (amongst female patients only)*				< 0.001
0	166 (44.4%)	60 (36.1%)	106 (63.9%)	
1	66 (17.7%)	15 (22.7%)	51 (77.3%)	
2	85 (22.7%)	–-	–-	
3 +	56 (15.0%)	–-	–-	
*History of prior cancer treatment*				0.018
Yes	121 (23.9%)	20 (16.5%)	101 (83.5%)	
No	385 (76.1%)	107 (27.8%)	278 (72.2%)	
*Documented fertility discussion*				< 0.001
Yes	254 (50.2%)	–-	–-	
No	252 (49.8%)	–-	–-	

Reported *p*-values are from chi-square tests or Fisher’s exact tests (indicated with *)

Values less than 5 are suppressed. Complementary suppression was utilized as necessary

This table has accounted and calculated for missing patient values

Occupation was categorized as follows: unemployed/unknown; business/finance/sales/management/legal (including accounting, investment, strategic planning, retail sales and sales representatives); computer/tech/architecture/engineering/science (related to IT, software and system administration, research and applied scientific fields); arts/media/sports; healthcare; service/construction/agriculture (customer service, manual labor, hospitality, food service, maintenance); administrative (clerical and office-based support roles) and student. Several occupation categories were combined due to small sample size

Referral rates significantly differed by occupation (*p* < 0.001). Referral rates were highest amongst students (referred 44.8%, not referred 55.2%) and lowest among unemployed patients (referred 10.4%, not referred 89.7%).

Most patients (96.2%) were insured with private insurance (80.4%), Medicaid (12.5%), and Medicare (3.1%). There were no differences in referral rates amongst insured and uninsured patients (*p* = 0.454) or by insurance type (*p* = 0.391). Single patients were more commonly referred (referred 30.7%; not referred 69.3%), followed by patients in a partnered relationship (referred 32.6%, not referred 67.4%) and married patients (referred 17.9%, not referred 82.1%) (*p* = 0.003). Referral rates differed by the number of living children for female patients (*p* < 0.001). Specifically, female patients without living children were more commonly referred (referred 36.1%; not referred 63.9%), followed by female patients with one child (referred 22.7%; not referred 77.3%).

The most common types of cancer were breast cancer (39.5%), lymphoma/leukemia (27.9%), and gynecologic cancer (14.6%). Referrals were based on oncologist triage and discretion. Referral rates were highest among lymphoma/leukemia patients (referred 34.8%, not referred 65.2%), gynecological cancer (referred 25.7%, not referred 74.3%), and breast cancer (referred 22.0%, not referred 77.0%), and lowest among those with cancer within the “other” category (referred 17.2%, not referred 82.8%) and sarcoma (referred 12.9%; not referred 87.1%) (*p* = 0.010). Patients with a prior history of cancer treatment (referred 16.5%, not referred 83.5%) had significantly lower referral rates compared to patients with no past cancer treatment (referred: 27.8%, not referred: 72.2%; *p* = 0.018). Finally, patients who had a documented fertility discussion had significantly higher referral rates than patients without (*p* < 0.001).

### Fertility preservation referral rates by cancer treatment type

Across cancer types, fertility preservation referral was not associated with planned gonadotoxic or teratogenic treatment (Table 2). Among patients with breast cancer, referral proportions were similar regardless of treatment (gonadotoxic 25.7% vs. 17.2%, *p* = 0.246; teratogenic = 22.6% vs. 24.1%; *p* = 0.976). For patients with gynecologic cancers, approximately one-quarter of patients in both those who did and did have planned gonadotoxic or teratogenic treatment were referred, with no significant differences observed (gonadotoxic *p* = 1.000; teratogenic *p* = 0.865). Referrals were more prevalent overall for patients with lymphoma/leukemia, but there were no significant differences in referral for patients with lymphoma/leukemia by planned treatment (gonadotoxic *p* = 0.057; teratogenic *p* = 0.958). For patients with sarcoma and patients with other cancers, small cell sizes largely preclude presentation of percentages, but no significant differences in referral were identified for gonadotoxic (other *p* = 0.370; sarcoma *p* = 0.335) or teratogenic (other *p* = 0.287; sarcoma *p* = 1.000).

**Table 2:** Fertility preservation referral rates by cancer treatment type

Cancer type	Gonadotoxic treatment			Teratogenic treatment		
	*Yes*	*No*	*p*-value	*Yes*	*No*	*p*-value
*Breast cancer*			0.246			0.976
Total	136 (68.0%)	64 (32.0%)		146 (73.0%)	54 (27.0%)	
Referred	35 (25.7%)	11 (17.2%)		33 (22.6%)	13 (24.1%)	
Not referred	101 (74.3%)	53 (82.8%)		113 (77.4%)	41 (75.9%)	
*Gynecologic cancer*			1.000*			0.865*
Total	55 (74.3%)	19 (25.7%)		28 (37.8%)	46 (62.2%)	
Referred	14 (25.5%)	5 (26.3%)		8 (28.6%)	11 (23.9%)	
Not referred	41 (74.5%)	14 (73.7%)		20 (71.4%)	35 (76.1%)	
*Lymphoma/leukemia*			0.057*			0.958
Total	129 (91.5%)	12 (8.5%)		96 (68.1%)	45 (31.9%)	
Referred	48 (37.2%)	–-		34 (35.4%)	15 (33.3%)	
Not referred	81 (62.8%)	–-		62 (64.6%)	30 (66.7%)	
*Other*			0.370*			0.287*
Total	17 (58.6%)	12 (41.4%)		20 (69.0%)	9 (31.0%)	
Referred	–-	–-		–-	–-	
Not referred	–-	–-		–-	–-	
*Sarcoma*			0.335*			1.000*
Total	50 (80.6%)	12 (19.4%)		29 (46.8%)	33 (53.2%)	
Referred	8 (16.0%)	–-		–-	–	
Not referred	42 (84.%)	–-		–-	–-	

Reported *p*-values for categorical variables are from chi-square tests or Fisher’s exact tests (indicated with *)

Values less than 5 are suppressed. Complementary suppression was utilized as necessary

### Predictors of FP referral by oncologist characteristics

Oncologist characteristics were not significantly associated with referral rates (Table 3). A greater, though not statistically significant, proportion of referrals were sent by female oncologists (27.6%) compared to male oncologists (20.6%; *p* = 0.107). There was no significant difference in referral by oncologist length since years of training (*p* = 0.096).

**Table 3:** Fertility preservation referral rates by oncologist characteristics

Oncologist characteristics	Referred (%)	Not referred (%)	*p*-value
*Gender*			0.107
Female	88 (27.6%)	231 (72.4%)	
Male	36 (20.6%)	139 (79.4%)	
*Years since training*			0.096
0–10	50 (21.0%)	188 (79.0%)	
11–20	23 (23.7%)	74 (76.3%)	
21–30	44 (32.1%)	93 (67.9%)	
31 +	7 (31.8%)	15 (68.2%)	

Reported *p*-values are from chi-square tests

### Predictors of referral for FP

After adjusting for covariates, male patients had higher odds of being referred than female patients (aOR = 2.01; 95% CI 1.01, 4.05, *p* = 0.048) (Table 4). Compared to patients aged 14–20, referral rates for patients aged 21–30 did not significantly differ (aOR = 0.78, 95% CI 0.24, 2.36, *p* = 0.659). However, patients aged 31–40 (aOR = 0.15, 95% CI 0.05, 0.47, *p* = 0.001) and 41–42 (aOR = 0.14, 95% CI 0.03, 0.61, *p* = 0.011) had significantly lower odds of being referred. Patients with previous chemotherapy had lower odds of referral than those who had not received chemotherapy (aOR = 0.46, 95% CI = 0.25, 0.83, *p* = 0.013).

**Table 4:** Predictors of referral for fertility preservation

Characteristics	aOR	95% CI	*p*-value
*Sex assigned at birth*			
Female	Referent	Referent	Referent
Male	2.01	1.01–4.05	0.048
*Age*			
14–20	Referent	Referent	Referent
21–30	0.78	0.24–2.36	0.659
31–40	0.15	0.05–0.47	0.001
41–42	0.14	0.03–0.61	0.011
*Partner status*			
Single	Referent	Referent	Referent
In a relationship	1.33	0.59–2.93	0.484
Married	0.64	0.37–1.10	0.110
*Race/ethnicity*			
Non-Hispanic White	Referent	Referent	Referent
Hispanic	0.98	0.38–2.42	0.962
Non-Hispanic Asian/Pacific Islander	0.61	0.24–1.43	0.274
Non-Hispanic Black	0.81	0.48–1.38	0.443
*Previous chemotherapy treatment*			
No	Referent	Referent	Referent
Yes	0.46	0.25–0.83	0.013
*Provider gender*			
Female	Referent	Referent	Referent
Male	0.90	0.48–1.68	0.747
*Provider years since training*			
0–10	Referent	Referent	Referent
11–20	1.39	0.71–2.70	0.329
21–30	2.89	1.50–5.62	0.002
31 +	1.71	0.56–4.88	0.326
*Cancer types*			
Breast	Referent	Referent	Referent
Gynecological	0.61	0.28–1.29	0.203
Leukemia/lymphoma	0.38	0.16–0.83	0.018
Other	0.35	0.09–1.18	0.110
Sarcoma	0.06	0.02–0.23	< 0.001

Acronyms: *aOR*, adjusted odds ratio; *CI*, confidence interval

Provider gender was not associated with referral (aOR = 0.90, 95% CI 0.48, 1.68, *p* = 0.747). Compared to patients with oncologists with 0–10 years since training, patients with oncologists with 21–30 years of experience had higher odds of being referred to FP (aOR = 2.89, 95% CI 1.50, 5.62, *p* = 0.002). There were no differences for patients with providers with 11–20 years (aOR = 1.39, 95% CI 0.71, 2.70, *p* = 0.329) or 31 years of training or more (aOR = 1.71, 95% CI = 0.56, 4.88, *p* = 0.326).

Patients with leukemia/lymphoma (aOR = 0.38, 95% CI = 0.16, 0.83, *p* = 0.018) and sarcoma (aOR = 0.06, 95% CI = 0.02, 0.23, *p* < 0.001) were less likely to be referred to FP compared to patients with breast cancer, but no difference was observed for gynecologic cancer (aOR = 0.61, 95% CI = 0.28, 1.29, *p* = 0.203) and cancers in the “other” category (aOR = 0.35, 95% CI = 0.09, 1.18, *p* = 0.110).

## Discussion

This study examined FP referrals among a group of diverse, reproductive-aged patients before the initiation of chemotherapy at a southeastern academic center. The low referral rate (22.5%) is consistent with several prior studies finding rates of less than 15%, which have not changed markedly over the last ten years [8–10]. This demonstrates a clear underutilization of FP services amongst reproductive-aged cancer patients, which has been attributed to various causes, such as lack of provider knowledge and clinic time constraints [12–15]. We identified significant associations between referral and biological sex, age, number of living children (among patients assigned female at birth), type of cancer, and provider years of training.

An important consideration in the interpretation of our study is inclusion of treatment that would require a significant delay in pregnancy. Although gonadotoxic treatment has a more immediate effect on reproductive organs, teratogenic treatment significantly impacts reproductive potential due to delays in planned conception and subsequent age-related infertility risks [25]. There are significant differences in fecundity every 1–2 years after women turn 35 years old with significant reductions after 40 years of age [26]. Infertility treatment also becomes less effective with advancing age [27]. Therefore, patients receiving teratogenic treatment with mandatory delay in pregnancy should be referred to REI for FP consultation as often as those receiving gonadotoxic therapy.

This study found that male patients were referred more often than female patients. Previous literature has conflicting results regarding sex differences in FP rates [28]. The absolute number of referred female patients was higher than male patients, which likely indicates that more female patients qualified for this study, possibly due to the complexity of cancer treatment and its effect on female reproduction. Chemotherapy tends to have a more pronounced effect on female gametes, as ovarian reserve decreases significantly with certain treatments. In contrast, male gametes tend to be more resilient to chemotherapy, with spermatogenesis frequently recovering after treatment [29].

The number of children that female patients had at time of cancer diagnosis also affected referral rates. Healthcare providers may view nulliparous patients as having different family planning priorities than those with children. Research has shown that patient wishes and provider assumptions for various family planning concerns, including existing and future family size, may not align [30]. Therefore, it is paramount to discuss FP with every patient, regardless of size of current family, to provide all patients with the opportunity to build the family they desire.

Additionally, patients with a history of prior cancer treatment were referred significantly less often than those who had no cancer history. FP may be a lower priority for Oncologists when treating patients with a prior cancer history, potentially due to treatments that may already have compromised their fertility. Although a diagnosis of POI would preclude FP, patients with lower, but still present, ovarian reserve would still benefit from consultation. We are not aware of any studies that explore how provider assumptions impact oncofertility referrals for patients with a history of cancer treatment. Of note, patients who had documented fertility discussions were more likely to be referred, potentially reflecting provider prioritization of and/or patients’ own advocacy for FP.

Despite making up a substantial portion of the study population, patients aged 31 years and older had lower referral rates compared to younger age groups. Although fertility potential begins to decline at 35 years old, fertility is still a priority for many patients in their 30 s and beyond. Additionally, women ages 30–40 years enter cancer treatment at higher risk of infertility and are more likely to need cryopreservation in the future. Students, patients with occupations in computer/tech/etc. and occupations in healthcare had the highest referral rates, while unemployed patients and patients in education had the lowest rates. Regardless of whether the patient chooses to pursue FP or not, the choice should be up to the patient, rather than the oncologist [31]. Patients value the counseling regardless of whether they proceed with treatment or not [32]. Referrals can empower patients with knowledge about reproductive options and bolster autonomy for future family planning.

In our study, patients with lymphoma/leukemia and sarcoma had lower odds of being referred for FP when compared to those with breast cancer and those with rare cancer types, such as sarcoma, had the lowest rate. These findings are consistent with prior literature showing different referral rates based on cancer type [18, 33]. Patients with sarcoma, specifically, have low rates of referral [34], despite high risks of infertility with treatment. Additionally, there is less data regarding the effects of fertility on some of the rarer diseases (e.g., multiple myeloma, aplastic anemia) included in this study, but emerging evidence suggests that some newer cancer treatment modalities (e.g., immunomodulators) may affect fertility [35], emphasizing that FP referrals should be considered with a diversity of treatments, consistent with ASCO recommendations [21].

While oncologist gender was not associated with referral, providers with 21–30 years of experience were more likely to refer patients than less experienced or more experienced physicians. To our knowledge, there is limited published data on Oncologist experience and the fertility care that they provide their patients. It may be that Oncology providers with 21–30 years of experience have been the most exposed to REI as a subspecialty given the advent of IVF in the early 1980 s and massive growth in cycle volume over the last three decades [36, 37]. It is also possible that Oncology physician training education has changed within those three decades, although we would have anticipated a greater emphasis on oncofertility with the passage of time. It is also possible that early-career physicians may feel less confident in initiating sensitive or emotionally charged fertility conversations compared to longer practicing colleagues. Another possibility is that younger physicians have greater reliance on standardized order sets. Lastly, even with modern training, the practical experience of watching the impact of fertility loss on cancer patients may accumulate over years. Thus, mid-career oncologists may have this more in mind to reinforce proactive FP referral.

This study did not find patient race and ethnicity to be a significant predictor of FP referrals. Other researchers have found mixed results regarding the impact of race and ethnicity on FP referrals [33, 38, 39]. We did have a diverse cohort of patients, with 39.3% identifying as non-Hispanic Black and 10.5% identifying as Asian or Pacific Islander. The racial and ethnic identities of the patients in this study reflect the overall community in which the hospitals are located. It is possible that physicians who care for a more diverse patient population are less likely to treat patients differently based on race and/or have done more implicit bias training to attempt to surmount disparities.

To increase FP referral rates, it is essential to implement standardized strategies that will integrate FP into routine oncology care. This includes education of oncology providers to address subjective biases on patient preferences. Providers should be equipped with knowledge and resources necessary to discuss FP options with their patients. Additionally, fostering interdisciplinary collaboration has been recommended to ensure consideration of all aspects of patient reproductive health [11, 12, 40, 41]. Referral systems for oncofertility are successful in improving referral rates and ensuring efficient communication to patients [42]. The integration of a smart tool into the EHR system is a promising solution that has not yet been substantially investigated, though it has been shown to have a positive impact in other medical specialties [43]. These tools provide Oncologists with real-time prompts for FP referrals, encouraging FP as part of the standard care pathway. Implementing referrals into routine oncology care will help establish FP as a consistent standard of care rather than a discretionary discussion for reproductive-aged cancer patients.

There were several limitations to the study. First, patients having an early oncology visit near the end of the study period may have documentation of FP discussions or referrals present in the EHR after the study period concluded. Consequently, some patients who were referred to REI may not be included in the study. Another limitation is that any discussions not documented in the EHR may limit the ability to completely represent FP discussions and referrals. Patients with cancers that had a small sample size (e.g., colon and aplastic anemia) were collapsed into a heterogeneous “other” category. This may have obscured meaningful factors influencing referrals within these subgroups. Lastly, the analysis of living children was restricted to female patients only.

This study’s strengths lie in its comprehensive approach to patient inclusion. By capturing a large cohort of patients directly from oncology records, we minimized selection bias that may arise from only analyzing those who actively seek out FP. This broad inclusion ensures a more representative sample of all eligible patients, not just those who received care from REI. Moreover, our detailed chart review allowed us to examine both patient and provider demographics, enabling an analysis of referral predictors that have not been extensively studied. The diversity of our cohort—spanning multiple cancer types, including rare malignancies treated with newer biologic agents—further strengthens the study’s relevance, as most prior research has focused primarily on breast cancer. Moreover, our use of multivariable analyses strengthens the study by accounting for multiple factors simultaneously, providing a more nuanced understanding of all of the factors that may influence FP referral patterns.

In conclusion, we identified patient and provider characteristics that were associated with FP referral for cancer patients. The integration of standardized referral tools into EHR systems could play a crucial role in addressing these gaps, streamlining the referral process and ensuring more consistent and equitable access to FP services. Observed disparities may be due to subjective Oncologist assumptions, emphasizing the need for a more inclusive and universal approach to counseling and referring patients to ensure equitable and consistent access to FP services.

## Data Availability

Not applicable.
